# Rapid Assessment of Ebola Infection Prevention and Control Needs — Six Districts, Sierra Leone, October 2014

**Published:** 2014-12-12

**Authors:** Ishani Pathmanathan, Katherine A. O’Connor, Monica L. Adams, Carol Y. Rao, Peter H. Kilmarx, Benjamin J. Park, Jonathan Mermin, Brima Kargbo, Alie H. Wurie, Kevin R. Clarke

**Affiliations:** 1 Epidemic Intelligence Service, CDC; 2 CDC Sierra Leone Ebola Response Team; 3 Sierra Leone Ministry of Health and Sanitation

As of October 31, 2014, the Sierra Leone Ministry of Health and Sanitation had reported 3,854 laboratory-confirmed cases of Ebola virus disease (Ebola) since the outbreak began in May 2014; 199 (5.2%) of these cases were among health care workers. Ebola infection prevention and control (IPC) measures are essential to interrupt Ebola virus transmission and protect the health workforce, a population that is disproportionately affected by Ebola because of its increased risk of exposure yet is essential to patient care required for outbreak control and maintenance of the country’s health system at large. To rapidly identify existing IPC resources and high priority outbreak response needs, an assessment by CDC Ebola Response Team members was conducted in six of the 14 districts in Sierra Leone, consisting of health facility observations and structured interviews with key informants in facilities and government district health management offices. Health system gaps were identified in all six districts, including shortages or absence of trained health care staff, personal protective equipment (PPE), safe patient transport, and standardized IPC protocols. Based on rapid assessment findings and key stakeholder input, priority IPC actions were recommended. Progress has since been made in developing standard operating procedures, increasing laboratory and Ebola treatment capacity and training the health workforce. However, further system strengthening is needed. In particular, a successful Ebola outbreak response in Sierra Leone will require an increase in coordinated and comprehensive district-level IPC support to prevent ongoing Ebola virus transmission in household, patient transport, and health facility settings.

Rapid needs assessments were conducted in Bombali, Moyamba, Port Loko, Pujehun, Tonkolili, and Western districts during October 1–5, 2014. These districts varied widely in Ebola case burden (8.3 cumulative confirmed cases per 100,000 population in Pujehun to 115.6 in Bombali [1]) and in the number of Ebola care facilities (one in Moyamba to 12 in Western). Data on existing IPC resources and activities currently under way as part of the Ebola response were collected in each district through key informant structured interviews and observations at health facilities using a standardized questionnaire.

The assessment team interviewed the district medical officer or a health management team representative to assess districtwide IPC activities, as well as a senior nursing or physician staff member at a convenience sample of 12 government-run referral health facilities. This included a district hospital as well as one to three Ebola “holding centers” per district (transitional care facilities where suspected Ebola patients are referred for diagnostic testing and supportive care until they can be transferred to a free-standing Ebola treatment unit for isolation and care), except in Tonkolili District where only the district hospital was visited. District hospitals are expected to screen for Ebola and properly isolate suspected patients while awaiting transfer to an Ebola treatment unit. Their Ebola isolation areas can become holding centers by default because of transportation delays and limited Ebola treatment unit bed availability. Standardized interview and assessment tools were based on World Health Organization Ebola infection prevention recommendations (2) and included questions on Ebola IPC response plans, procedures, facilities, staffing, transportation teams, and supplies. Interviewee responses were recorded by hand and compiled for qualitative review. Assessment team members were doctoral-level international health professionals from CDC. They did not enter active Ebola care wards to directly observe IPC systems or practices.

Widespread gaps in IPC systems and resources critical for Ebola prevention and response were identified through interviews with key informants in all six districts visited (Table). None of the districts had dedicated infection control focal persons or supervisors within district health management structures to coordinate IPC activities and conduct routine quality assurance at the time of the rapid assessment. Furthermore, no IPC standard operating procedures existed at facility, district, or national levels for proper screening, isolation, care, and transport of suspected, probable, and confirmed Ebola patients.

Ebola screening procedures at all facilities visited were inadequate to facilitate appropriate triage and separation of patients suspected of having Ebola from those not suspected of having Ebola. Overall, there was a need for a standard routine screening protocol to minimize case misclassification, screening positioning at the initial access-controlled point of entry, and proper use of PPE among screeners. PPE supplies were reported to be insufficient for patient care and transport activities in every district, with larger gaps for rural facilities, clinics, and ambulance teams. Other deficiencies in supplies and infrastructure included lack of running water, working incinerators for burning disposable waste, chlorine, and blood collection supplies. A detailed list of district-specific needs was compiled for presentation to key national stakeholders.

Key informants reported that the availability of hospital and holding center staff competent in IPC practices also was inadequate. The shortage was compounded by deaths of health care workers from Ebola infection and workforce attrition resulting from delays in receiving hazard pay and from staff fatigue (in two districts, medical officers responsible for operating Ebola isolation wards and ensuring staff adherence to IPC had not had a day off in over 2 months). However, the biggest barrier to adequate staffing was that IPC training and mentoring had not yet been uniformly delivered to staff members before the opening of the Ebola care facility. Only three of six districts reported that basic training had been provided to facility health care workers, including PPE use. In two districts, basic training had not been provided to most staff members, although PPE was being used. Ambulance teams and cleaners were reported to have undergone formal IPC training less consistently than burial teams and laboratory technicians, and staff members at peripheral health units (community clinics in Sierra Leone) were not yet routinely trained to safely screen for or isolate persons suspected with Ebola before transport to Ebola care facilities. Overwhelmingly, refresher IPC training and mentorship were desired, even in districts where IPC training activities had taken place.

Finally, delays in Ebola patient transportation and reporting of laboratory results hindered the separation of confirmed Ebola patients from suspected Ebola patients in holding centers, or from their families and communities. In areas distant from Ebola diagnostic laboratories, sample result turnaround time varied and sometimes took as long as 1 week. In two districts, home care was occurring regularly because of delays in patient transport systems and Ebola care bed availability, but without clear guidance for families on how this could be done safely. In all assessed districts, additional all-terrain vehicles and fuel were urgently needed for burial and ambulance teams, as well as specimen transport. No standard operating procedures were readily available for cleaning and decontamination of these vehicles which, in conjunction with limited training, improper use of PPE, and poor separation between clean and contaminated areas in the vehicles, put transport teams and potentially uninfected but suspected Ebola passengers at risk for infection.

## Discussion

Based on these findings and key stakeholder input, priority IPC actions for the Ebola response in Sierra Leone were recommended. The Ministry of Health and Sanitation and international Ebola response partners have developed IPC protocols for care and transport procedures for implementation at the district and facility levels. They are increasingly procuring and organizing necessary supplies and support, and prioritizing growth of laboratory and Ebola treatment capacity. Given the lack of a preexisting infection control cadre and the overwhelming need for well-trained staff at all facility levels, the team recommended the rapid establishment of a large-scale Ebola treatment and IPC training program adapted to the varied health responder workforce. This program now exists and is being scaled up with international partner support. IPC training and delivery of PPE and other supplies to 1,185 peripheral health units is under way with technical support from CDC. Finally, monitoring and evaluation through a comprehensive Ebola IPC quality assurance system, including core IPC metrics, is planned and is expected to reinforce prevention efforts.

Additionally, national Ebola IPC coordination is ensuring that identified IPC gaps are addressed rapidly, correctly, and efficiently. Lead IPC response partners are coordinating standard operating procedure implementation, providing comprehensive IPC assessment and remediation of deficits at health care facilities, implementing routine IPC monitoring, and supporting facility-level commodity management. Strict administrative controls of patient screening and care in facilities continue to be needed to prevent infection of health care workers, uninfected patients, and visitors. Trained IPC specialists embedded within health care facilities and at the district level are recognized as critical to providing oversight of IPC strategy implementation; efforts to train and place these staff are underway.

Moving forward, ongoing IPC refresher training and corrective IPC practice reinforcement will be needed at the facility level following initial training. Ambulance transport capacity should be increased with improved IPC protocols to avoid transportation-related infections and, if care is to take place increasingly in homes, a clear protocol and strategy for this is imperative to prevent further community transmission. Finally, consensus criteria should be established both for IPC standards to be met before Ebola care facility opening and for closing facilities that fail to meet minimum standards.

Results from this rapid assessment were limited by time constraints, absence of assessment in Ebola patient care areas, and potential response bias from interviews administered to district-level stakeholders. In addition, the assessment team had varied success with key informant availabilities and the number of sites visited. Nevertheless, the assessment provides rapid insight into current IPC practices and preparedness in communities, patient transport, and health facility settings. An increasingly coordinated and comprehensive IPC program with district and health facility level support is urgently needed to prevent Ebola in districts where the prevalence is low and to strengthen the existing IPC response in areas with high prevalence of Ebola.

What is already known on this topic?

Sierra Leone continues to have a large number of Ebola cases. Ebola infection prevention and control (IPC) measures are essential to interrupt Ebola virus transmission and protect the health workforce.

What is added by this report?

A rapid needs assessment of six districts in Sierra Leone identified widespread gaps in IPC systems and resources critical for Ebola prevention and response in communities, patient transport, and health facility settings. In particular, there were shortages of trained staff members, personal protective equipment, safe transport, and standardized IPC protocols.

What are the implications for public health practice?

Based on rapid assessment findings and key stakeholder input, priority IPC actions for the Ebola response in Sierra Leone were recommended. A successful response will require an increase in coordinated and comprehensive district-level IPC support to prevent ongoing Ebola virus transmission in the country.

## Figures and Tables

**TABLE:** Infection prevention and control (IPC) response assessment as reported by district medical officers and stakeholders — six districts, Sierra Leone, October 1–5, 2014

	Bombali	Moyamba	Port Loko	Pujehun	Tonkolili	Western
Ebola cumulative incidence per 100,000 population	115.6	34.5	99.8	8.3	48.3	88.7
IPC standard operating procedures in place	No	No	No	No	No	No
IPC practitioner on staff	No	No	No	No	No	No
Proper screening by protocol	No	No	No	No	No	No
Recommended personal protective equipment available*	No	No	No	No	—†	No
Adequate staff	No	No	No	No	No	No
**Persons with any IPC training** §						
Health care workers	Yes¶	Yes¶	No	No	Yes¶	No
Burial teams	Yes¶	Yes¶	—	Yes¶	—	Yes¶
Ambulance teams	No	No	No	No	—	No
Cleaners	No	Yes¶	Yes¶	No	Yes¶	No
Laboratory technicians	Yes¶	Yes¶	Yes¶	Yes¶	—	—
Refresher training desired	Yes	Yes	Yes	Yes	Yes	Yes
No. of ambulances (% coverage**)	5 (38%)	1 (7%)	3 (27%)	1 (8%)	—	6
Reported no. of days until return of Ebola laboratory results	2–7	2¶	2–5	2¶	2–6	2–3
Care in homes occurring††	Rare¶	Rare¶	50–100	Rare¶	—	Many

* Recommended refers to appropriate quantity and quality.

† Information not available.

§ IPC training was only counted if it included personal protective equipment procedures and participation by the majority of staff members.

¶ Response needs being met.

** Percentage coverage of chiefdoms (assuming goal of one ambulance per chiefdom). There are no chiefdoms in the Western District.

†† Estimated number of known Ebola cases remaining in homes.
